# Bcl-2 Inhibits the Innate Immune Response during Early Pathogenesis of Murine Congenital Muscular Dystrophy

**DOI:** 10.1371/journal.pone.0022369

**Published:** 2011-08-05

**Authors:** Sheila Jeudy, Katherine E. Wardrop, Amy Alessi, Janice A. Dominov

**Affiliations:** Boston Biomedical Research Institute, Watertown, Massachusetts, United States of America; Ohio State University, United States of America

## Abstract

Laminin α2 (*LAMA2*)-deficient congenital muscular dystrophy is a severe, early-onset disease caused by abnormal levels of laminin 211 in the basal lamina leading to muscle weakness, transient inflammation, muscle degeneration and impaired mobility. In a *Lama2*-deficient mouse model for this disease, animal survival is improved by muscle-specific expression of the apoptosis inhibitor Bcl-2, conferred by a MyoD-hBcl-2 transgene. Here we investigated early disease stages in this model to determine initial pathological events and effects of Bcl-2 on their progression. Using quantitative immunohistological and mRNA analyses we show that inflammation occurs very early in *Lama2*-deficient muscle, some aspects of which are reduced or delayed by the MyoD-hBcl-2 transgene. mRNAs for innate immune response regulators, including multiple Toll-like receptors (TLRs) and the inflammasome component NLRP3, are elevated in diseased muscle compared with age-matched controls expressing *Lama2*. MyoD-hBcl-2 inhibits induction of TLR4, TLR6, TLR7, TLR8 and TLR9 in *Lama2*-deficient muscle compared with non-transgenic controls, and leads to reduced infiltration of eosinophils, which are key death effector cells. This congenital disease model provides a new paradigm for investigating cell death mechanisms during early stages of pathogenesis, demonstrating that interactions exist between Bcl-2, a multifunctional regulator of cell survival, and the innate immune response.

## Introduction

Muscular dystrophies are degenerative neuromuscular diseases characterized by progressive loss of muscle structure and function that impairs mobility, reduces quality of life, and can lead to early lethality. There are at least 40 types of muscular dystrophies, varying in onset and severity, which are caused by genetic changes affecting proteins required for normal muscle function [1]. Congenital muscular dystrophies are early onset diseases with muscle weakness occurring at or shortly after birth. They are typically caused by abnormal expression of proteins required for structural and signaling linkages between muscle cells and the extracellular matrix surrounding them [2]. Laminin α2 (*LAMA2*)-deficient congenital muscular dystrophy (Muscular Dystrophy, Congenital Merosin-deficient Type 1A, (MDC1A)) is a severe form of this type of disease. Laminins are trimeric proteins comprised of an α, β and γ chain. Laminin-211 (α2β1γ1 or merosin) is normally the most prevalent form in the basal lamina around muscle fibers [3]. Laminin-211 interacts with α-dystroglycan and α7β1 integrin on the muscle fiber, and integrity of these interactions is critical for muscle function and survival. In MDC1A, caused by insufficient levels of laminin α2 chain [4], there is early onset weakness, a transient phase of inflammation, loss of muscle fibers and poor muscle regeneration, leading to irregular muscle fibers, fat deposition and fibrosis.

Degeneration of muscle in muscular dystrophies is typically accompanied by inflammatory responses that can vary in magnitude and temporal progression and play a significant role in the physiology of the diseased muscle. As recently reviewed [5], there is a dynamic relationship between skeletal muscle cells and cells of the immune system, which routinely survey tissues for abnormalities, respond quickly to destroy and remove damaged elements and promote muscle repair.

Using a *Lama2*-deficient mouse model (*Lama2^dy-W^* [6]), we found that expression of the proinflammatory cytokines TNFα and IL-1β is elevated in *Lama2*-mutant muscles within 7 days after birth, prior to widespread muscle degeneration [7]. *Lama2*-deficient muscle also had increased expression of tenascin C (TN-C), a matrix glycoprotein upregulated in pathological conditions such as inflammation, as well as reduced expression of lymphatic vessel endothelial hyaluronan receptor-1 (LYVE-1) in lymphatic capillaries within the muscles [7]. LYVE-1 is specifically found in lymphatic endothelial cells and is rapidly degraded when these cells are exposed to TNFα [8], therefore changes in both TN-C and LYVE-1 could result from elevated inflammatory cytokines present in 7 day-old *Lama2*-deficient muscles. Between 7 and 14 days of age, inflammation in *Lama2*-deficient mouse muscle is accompanied by apoptotic and necrotic fiber degeneration. These features in the mouse model correlate with the early, transient inflammation and fiber degeneration in human MDC1A [9], [10], [11].

Accumulating evidence indicates that ‘presymptomatic’ activation of inflammatory pathways is a common feature of other, later-onset forms of muscular dystrophy as well, including those caused by deficiencies in dysferlin or dystrophin [12], [13]. The mechanisms through which defects in muscle structure lead to activation of inflammatory pathways have not been determined. It is likely, however, that this process involves regulation of the innate immune system, which is poised to quickly respond to abnormal macromolecules within damaged tissue. The *Lama2*-deficient mouse model, with its early-onset inflammation, provides an ideal system to study the role of the innate immune pathways in muscle pathogenesis.

The innate immune response is an inherent mechanism, functional at birth, which protects organisms from pathogens. It also plays a broader role in pathologies related to endogenous tissue damage, inflammation and autoimmune diseases [14]. One general mechanism of innate immunity relies on recognition of aberrant molecular patterns within diseased or damaged tissues. Molecules with pathogen-associated molecular patterns (PAMPs) (from microorganisms) or damage-associated molecular patterns (DAMPs) (from endogenous tissues) are recognized by specific receptors on immune system cells (e.g. macrophages) such as Toll-like receptors (TLRs) [15], [16]. Upon binding to DAMP/PAMP ligands, TLRs induce NF-κβ activation and subsequent TNFα and pro-IL-1β synthesis. IL-1β release from cells requires additional pro-IL-1β processing by caspase-1, which occurs within multi-component inflammasome complexes containing the “nucleotide-binding domain leucine-rich repeat containing” receptor (NLR) protein NLRP3 [17]. NLRP3 (NALP3, cryopyrin) is critical for propagation of innate inflammatory signals. These innate pathways contribute significantly to the development of inflammatory disorders [16], kidney disease [15], and metabolic diseases [17], and inflammasome components are upregulated in dysferlin-deficient muscular dystrophy [18].

There are at least 13 mammalian TLRs that recognize numerous specific DAMP/PAMP ligands to promote inflammation in damaged tissues [14], [15]. DAMP ligands [and respective TLRs] include hyaluronan fragments, heat shock proteins (HSPs), High Mobility Group Box 1 Protein (HMGB1), biglycan, uric acid, lipoproteins [TLR2 or TLR4], mRNA, double-stranded RNA [TLR3], fibrinogen, heparan sulfate, fibronectin [TLR4], single-stranded RNA [TLR7 or TLR8], and unmethylated CpG motif DNA [TLR9]. TLR1 and TLR6 act as dimers with TLR2. Some TLRs (e.g. TLR1, TLR2, TLR4, TLR6) are localized at the plasma membrane and recognize extracellular ligands, while others (TLR3, TLR7, TLR8, TLR9) are intracellular receptors, associated with endoplasmic reticulum (ER) and endosome membranes and recognize intracellular ligands.

In previous studies using *Lama2*-deficient mice, we found that muscle-specific overexpression of the apoptosis inhibitor Bcl-2 (via MyoD-hBcl-2 or MRF4-hBcl-2 transgenes) or systemic inactivation of the pro-apoptotic protein Bax (using *Bax^−/−^* mice) improved animal size and survival [19], [20], demonstrating that altering expression of intrinsic apoptosis pathway regulators can dramatically affect long term pathology. We sought to determine whether altering Bcl-2 levels could also modulate the earliest events in pathogenesis, focusing on the early inflammation in this disease and elements of innate immunity.

Here we report that specific elements of the innate immune response are significantly induced in 7 day-old *Lama2*-deficient muscles. These include mRNAs for TLR1, TLR2, TLR4, TLR6, TLR7, TLR8, TLR9, and NLRP3, along with increased eosinophil infiltration and eotaxin-1 expression. Muscle-specific Bcl-2 expression reduces the initial expression of TN-C in *Lama2*-deficient muscles, while some other indices of pathology (macrophage/neutrophil/monocyte infiltration, LYVE-1 expression) are not affected. Moreover, muscle-specific Bcl-2 expression significantly inhibits the induction of TLR4, TLR6, TLR7, TLR8, and TLR9 and reduces eosinophil infiltration. Together, our data demonstrate that specific regulators of the innate immune response are induced in neonatal muscles as a consequence of the disruption in normal muscle cell-extracellular matrix interactions, and this process can be modulated by Bcl-2.

## Materials and Methods

### Animals

All procedures involving mice were approved by the Boston Biomedical Research Institute Animal Care and Use Committee (permit number 01-07) and followed guidelines of the US Public Health Service Policy on Humane Care and Use of Laboratory Animals and the USDA Animal Welfare Act. Wild-type C57BL/6J mice (Jackson Laboratories, Bar Harbor, ME, USA), *Lama2*-deficient mice (*Lama2^dy-W^*) with a targeted *lacZ* insertion disrupting normal laminin α2 expression [6], and MyoD-hBcl-2 transgenic mice that overexpress human Bcl-2 specifically in skeletal muscles, driven by a mouse MyoD promoter [19], [20], were maintained in a pathogen-free, temperature controlled environment. *Lama2*-deficient mice were generated by interbreeding heterozygotes (*Lama2^+/−^*) because of early lethality in *Lama2^−/−^* animals. To generate *Lama2^−/−^* mice that carried the MyoD-hBcl-2 transgene, *Lama2^+/^* ^−^ mice were bred with transgenic MyoD-hBcl-2 mice to generate F1 progeny that were *Lama2^+/−^*; *MyoD-hBcl-2^+/^* ^−^. These *Lama2^+/−^*; *MyoD-hBcl-2^+/^* ^−^ mice were then bred to *Lama2^+/^* ^−^ mice to generate the F2 progeny used in these experiments. In this way, all *Lama2^+/+, +/− or −/^* ^−^ animals used in experiments involving the MyoD-hBcl-2 transgene were from the F2 generation and carried either a single copy of the transgene or no transgene. Mice were genotyped using PCR as described [20].

Muscle samples for RNA or protein analysis were collected at various ages and immediately snap frozen on dry ice. Upper hindlimb muscle samples included all muscles surrounding femur except the quadriceps. For immunohistological analyses, muscles or intact lower hindlimbs were embedded in O.C.T. freezing compound (Tissue-Tek, Sakura-Finetek, Torrence, CA, USA), then quickly frozen in isopentane chilled with liquid nitrogen.

### Immunostaining and TUNEL staining

Frozen tissues were cryosectioned (10 µm sections) and immunostained to analyze protein expression as described [7]. Primary antibodies used were rat anti-mouse TN-C (diluted 1/500, clone MTn-12, T3413, Sigma, St. Louis, MO, USA,), rabbit anti-mouse LYVE-1 (1/500, 14917, Abcam, Cambridge, MA, USA), rat anti-mouse major basic protein (MBP) (1/1000, clone MT-14-7-3, from J.J. Lee, Mayo Clinic) and Alexa Fluor 488-labeled rat anti-mouse CD11b (1/500, M1/70, 557672, BD Pharmingen, San Diego, CA, USA). Secondary antibodies used were Alexa Fluor 488 goat anti-rabbit IgG (1/1000, A11070)), Alexa Fluor 594 goat anti-rabbit IgG (1/1000, A11072), Alexa Fluor 546 goat anti-mouse IgG (1/1000, A11018,) all from Invitrogen (Carlsbad, CA, USA) and Cy3 goat anti-rat IgG (1/2000, 112-165-006, Jackson Immunoresearch, West Grove, PA, USA).

TUNEL assays were used to identify apoptotic cells in muscle tissue. Lower hindlimbs sections were fixed with 4% paraformaldehyde and TUNEL stained using an In Situ Cell Death Detection Kit (Roche Applied Science, Indianapolis, IN, USA) following the manufacturer's protocol. TUNEL stained samples were then immunostained to identify the sarcolemma or basal lamina in order to distinguish apoptotic nuclei associated with muscle fibers from non-muscle nuclei in the interstitial space. For 7 day-old muscles, rabbit anti-human dystrophin (diluted 1/1000, 15277 Abcam, Cambridge, MA, USA) was used to label the sarcolemmal membrane. At 14 days of age, however, there was significant loss of dystrophin expression from diseased muscle membranes. Therefore rabbit anti-mouse (pan)-laminin, (1/100, L9393, Sigma, St. Louis, MO, USA), which recognizes other laminin isoforms present in *Lama2*-deficient muscle, was used to label the basal lamina around fibers. Appropriate Alexa Fluor 488 or 594 secondary antibodies (above) were used to visualize the muscle fiber-associated antigens and to distinguish TUNEL(+) nuclei within or outside of muscle fibers. Muscles in the TA/EDL and gastrocnemius/soleus regions of each limb were imaged and the number of TUNEL(+) cells within and outside of the basal lamina or sarcolemma were quantified.

Samples were imaged using a Leica DMR microscope and imaging system with Leica IM50 Image Manager software. Minor linear brightness and contrast adjustments were made to some images using Adobe Photoshop, with adjustments applied to the entire image and equivalently applied to each sample within an experimental set. Morphometric analysis of antigen expression was done using NIH Image J software.

### Western Blot Analysis

Proteins were extracted from frozen muscle samples and protein was analyzed on western blots as previously described [20] (10–50 µg protein/lane, equal amounts loaded/lane on each blot). Antibodies used were: rabbit anti-human Apaf-1 (apoptotic protease-activating factor-1, diluted 1/1000, AB16941, Chemicon, Billerica, MA, USA), rabbit anti-human XIAP (X-linked inhibitor of apoptosis, 1/1000, 2042, Cell Signaling Technology, Danvers, MA, USA), rabbit anti-human FLIP (FLICE-inhibitory protein, 1/1000, ab8421, Abcam, Cambridge, MA, USA), and rabbit anti-human ARC (apoptosis repressor with caspase recruitment domain, 1/1000, 160737, Cayman Chemical, Ann Arbor, MI, USA). Each blot was simultaneously probed with mouse anti-rabbit GAPDH (1/1000, 10R-G109a, Fitzgerald, Acton, MA, USA) for normalization of protein loading. Secondary antibodies used for detection were Alexa Fluor 680 goat anti- rabbit IgG (1/10,000, A21109, Invitrogen, Carlsbad, CA, USA) and IRDye 800 goat-anti mouse IgG (1/10,000, 610-132-121, Rockland, Gilbertsville, PA, USA). Blots were scanned using a LI-COR Odyssey Infrared Imaging System to visualize protein expression.

### RNA Expression

RNA was extracted from frozen muscle tissues, treated with DNAse as described [7] then reverse transcribed using a High Capacity cDNA Reverse Transcription Kit (Applied Biosystems, Carlsbad, CA, USA) following the manufacturer's protocol. Resultant cDNAs were analyzed by quantitative PCR (QPCR) using triplet reactions per sample. Eotaxin-1 and glyceraldehyde 3-phosphate dehydrogenase (GAPDH) were analyzed in TaqMan probe QPCR assays as described [7], using the following primer/probe sets (listed 5′-3′): eotaxin-1 (CCL11, NM_011330) forward TCAAGACCAGGTTGGGCAAAGA, reverse GGTTAGTGTCAAGAGAGGAGGTT, probe 5′FAM- AAGAAGTGGGTCCAGGATGCCACAAA-3′TAMRA; GAPDH (NM_008084.2) forward TGTGTCCGTCGTGGATCTGA, reverse CCTGCTTCACCACCTTCTTGA, probe 5′FAM-CCTGGAGAAACCTGCCAAGTATGA-3′TAMRA. SYBR Green QPCR assays (DyNAmo HS SYBR Green Q-PCR Kit, Finnzymes/ThermoFisher, Waltham, MA, USA) were used for other genes (primers in Table 1) using the following PCR conditions: 95°C 15 min., 45 cycles of (94°C 10 sec., 60°C 30 sec., 72°C 30 sec.), 72°C 5 min., followed by a 65°C–95°C melt curve to confirm the amplification of single products for each reaction. Separate reactions without reverse transcriptase confirmed that RNA samples did not contain DNA that interfered with the PCR analysis. Relative mRNA levels, expressed relative to a normal wild-type sample within each data set, were calculated after normalizing to GAPDH levels in each sample.

**Table 1:** Primers Used for SYBR Green Quantitative RT-PCR Assays.

Gene	Accession Number	Forward Primer (5′ – 3′)	Reverse Primer (5′- 3′)	Amplicon Size (bp)	Primer Spans Exon Junction
TLR1	NM_030682.1	CCGTGATGCACAGCTCCTTGGTT	GGGTATAGGACGTTTCTGTAGGGGT	109	Yes - Reverse
TLR2	NM_011905.3	CTAGGCTGGTGCCCAGATGGCTAG	CCTGCCCGGAGCCTAGGAGTC	70	Yes - Reverse
TLR3	NM_126166.4	TAAAGAGTTCTCCCCGGGGTGTTTCC	GGTGGGGGTTCAGTTGGGCG	83	Yes- Forward
TRR4	NM_021297.2	CCCTGCATAGAGGTAGTTCC	GCCATGCCATGCCTTGTCTTCA	219	Yes - Forward
TLR6	NM_011604.3	ACCGTCAGTGCTGGAAATAGAGCTT	AGGCCAGGGCGCAAACAAAGT	142	Yes - Forward
TLR7	NM_133211.3	AGGCTCTGCGAGTCTCGGTT	GGAGCCAAGGACATCTTTCTTGAAGGC	99	Yes - Reverse
TLR8	NM_133212.2	GAACATGGAAAACATGCCCCTCA	TGCAATCACAAGGGAGTTGTGCCT	145	Yes - Forward
TLR9	NM_031178.2	CTGAGAGACCCTGGTGTGGAACA	CGACGGAGAACCATGTTGGGAGA	96	Yes - Reverse
NLRP3	NM_145827.3	TGCCTGTTCTTCCAGACTGGTGA	CACAGCACCCTCATGCCCGG	144	Yes - Forward
GAPDH	NM_008084.2	ACCCAGAAGACTGTGGATGG	CACATTGGGGGTAGGAACAC	170	No

### Statistics

Statistical significance was calculated using unpaired t-tests, or nonparametric Mann-Whitney tests and Prism 4.0c statistical analysis software (GraphPad Software, La Jolla, CA, USA). All data presented are mean values ± SEM. All statistically significant differences in data values are noted (*) as described in figure legends.

## Results

### Muscle-specific Bcl-2 expression reduces early expression of TN-C in *Lama2*-deficient muscle

**Figure 1:**
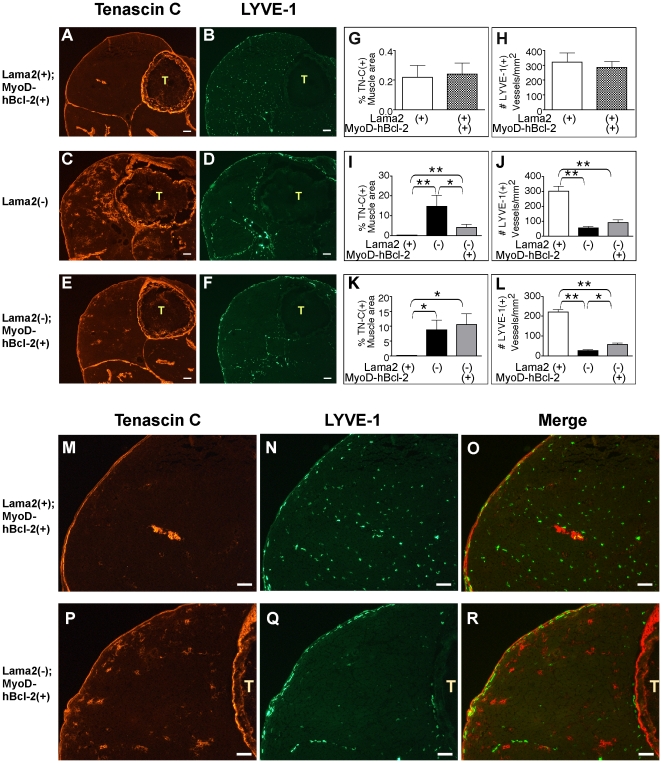
Expression of TN-C, but not LYVE-1 is affected by MyoD-hBcl-2 in 7 day-old *Lama2*-deficient muscle. (**A–F**, **M–R**) Hindlimbs were immunostained with antibodies to TN-C (**A**, **C**, **E**) and LYVE-1 (**B**, **D**, **F**). (**A**) MyoD-hBcl-2 transgenic *Lama2^+/−^* mice had strong staining of epimysium and tendons but negligible levels of TN-C around muscle fibers, similar to non-transgenic normal mice at this age. (**C**) TN-C expression was elevated around *Lama2^−/−^* muscle fibers, but this was reduced in MyoD-hBcl-2 transgenic *Lama2^−/−^* muscles (**E**). (**B**) *Lama2^+/−^*; *MyoD-hBcl-2^+/−^* mice had a normal punctate pattern of LYVE-1 expression, marking lymphatic capillaries within the muscle bed and interstitium as in non-transgenic normal mice. (**D**) LYVE-1-expression was reduced in capillaries within the muscle bed of *Lama2^−/−^* mice, and this was not affected by the MyoD-hBcl-2 transgene at 7 days of age (**F**). (**G and H**) Quantitation of TN-C and LYVE-1 expression in 7 day-old *Lama2-*expressing (*Lama2(+)*: *Lama2^+/+ or +/−^*) muscle either with or without MyoD-hBcl-2 transgene showed there was no effect of transgene on these markers in normal muscle (n = 4–5, p>0.05). (**I and J**) Quantitation of TN-C and LYVE-1 expression in 7 day-old *Lama2(+) and Lama2(−) (Lama2^−/−^)* muscle with or without MyoD-hBcl-2 transgene (n = 7–9). (**K and L**) Quantitation of TN-C and LYVE-1 in 14 day-old muscles (n = 3–4) (* p<0.03, ** p<0.001). (**M–R**) Higher magnification images of muscle samples show that reduced LYVE-1 expression occurs more broadly throughout the muscle bed of *Lama2*-deficient mice than elevated expression of TN-C. (**M**, **N**) MyoD-hBcl-2 transgenic *Lama2^+/−^* muscle in panels 1A and 1B showing normal expression of TN-C and LYVE-1, respectively. (**P**, **Q**) MyoD-hBcl-2 transgenic *Lama2^−/−^* muscle in panels 1E, and 1F, showing elevated TN-C and reduced LYVE-1 staining. (**O**) and (**R**) are merged images. (Scale bars in all images: 100 µM, T: tibia).

Early pathology in mice with *Lama2*-deficient muscular dystrophy is marked by increased expression of TN-C in the extracellular matrix around muscle fibers, and decreased expression of LYVE-1 in lymphatic capillaries within the muscles [7]. We bred *Lama2^+/−^* mice with MyoD-hBcl-2 transgenic mice that express human Bcl-2 specifically in skeletal muscle as done previously [19], [20] to determine the effects of muscle-specific Bcl-2 expression on early pathogenesis, focusing specifically on pathology one to two weeks after birth. At 7 days of age, MyoD-hBcl-2 transgenic mice that express *Lama2* (*Lama2(+)*: *Lama2^+/+^* or *Lama2^+/−^*) had minimal levels of TN-C in the matrix around fibers, and normal levels of LYVE-1 in the numerous lymphatic capillaries present within the muscle bed (Figures 1A, B, M, N, O) similar to normal patterns observed previously in non-transgenic *Lama2(+)* at this age [7]. Quantitative analysis determined that MyoD-hBcl-2 expression did not affect expression of these markers in *Lama2(+)* muscles (Figures 1G, H). TN-C expression was markedly increased in *Lama2(−)* (*Lama2^−/−^*) muscles at this age (Figure 1C). However, MyoD-hBcl-2 transgenic *Lama2(−)* mice expressed significantly less TN-C than non-transgenic cohorts (Figures 1E, I), indicating that signals driving its expression, perhaps related to inflammation, were reduced by Bcl-2 expression. This effect was transient however, and by 14 days of age, MyoD-hBcl-2 transgenic *Lama2(−)* muscle also expressed significant levels of TN-C (Figure 1K), affecting most of the muscles within the limb. Consistent with previous results [7], LYVE-1 expression was reduced in lymphatic endothelial cells within *Lama2(−)* muscle tissues, but unlike TN-C expression, this was not affected by MyoD-hBcl-2 expression at 7 days of age (Figures 1D, F, J,Q). Higher magnification images of these samples (Figures 1M–1R) show that, as previously reported [7], a broader area of muscle had reduced LYVE-1 expression as compared with elevated TN-C expression. LYVE-1 expression was slightly higher in transgenic *Lama2(−)* muscle tissue at 14 days of age (Figure 1L). Thus not all aspects of early pathology that could be affected by inflammation were reduced by muscle-specific Bcl-2 transgene expression.

### Muscle-specific Bcl-2 effects on prevalence of apoptotic cells, apoptosis regulators and inflammatory cell infiltration

**Figure 2:**
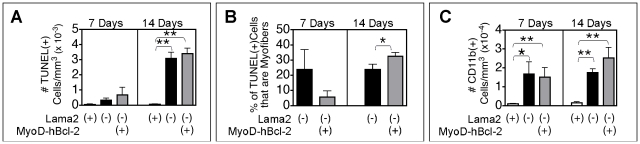
Effects of MyoD-hBcl-2 on the prevalence of apoptotic and inflammatory cells in *Lama2*-deficient muscles. (**A**) Lower hindlimb muscles from 7 and 14 day-old mice were TUNEL stained to detect apoptotic nuclei within muscle tissues (*Lama2(+)*: *Lama2^+/+ or +/^* ^−^, *Lama2(−)*: *Lama2^−/−^*). There were no TUNEL(+) cells in *Lama2(+)* muscle at 7 days (n = 2 mice) and few in *Lama2*-deficient muscle (n = 4 or 5 mice, *Lama2(−)* without or with transgene respectively), but these increased in number by 14 days, and the MyoD-hBcl-2 transgene did not affect this (n = 5–8 mice). (**B**) These samples were additionally immunostained to distinguish apoptotic muscle cells (nuclei within the basal lamina or sarcolemma) from interstitial cells. Of the few apoptotic cells present in *Lama2(−)* tissue at 7 days, there was a trend that fewer were muscle cells in the MyoD-hBcl-2 transgenic tissue, but at 14 days, significantly higher proportions of the apoptotic cells in transgenic tissue were muscle cells. (**C**) CD11b(+) cells (macrophages, neutrophils, monocytes) were present in *Lama2*-deficient quadricep muscles at both 7 and 14 days and MyoD-hBcl-2 transgene did not affect their prevalence. (n = 3–7 mice, *p<0.04, **p<0.003).

We examined 7 and 14 day-old muscle for other aspects of pathology, including markers of apoptosis and inflammation. Only a small number of apoptotic (TUNEL(+)) cells were present in *Lama2(−)* limb muscles at 7 days of age (Figure 2A), and these were mostly interstitial cells and not myofibers. The MyoD-hBcl-2 transgene did not significantly affect the number of TUNEL(+) cells. However, of the few TUNEL(+) cells present, there was a trend in MyoD-hBcl-2 transgenic mice for fewer to be myofiber cells than interstitial cells when compared with non-transgenic *Lama2(−)* mice at this age. For example, 38% of the 7 day-old *Lama2(−)* gastrocnemius/soleus and TA muscle regions examined (n = 8 from 4 mice) had some TUNEL(+) myofibers, while only 22% of the cohort MyoD-hBcl-2 transgenic *Lama2(−)* muscles (n = 9 from 5 mice) contained TUNEL(+) myofibers. The proportion of the TUNEL(+) cells that were myofibers in these muscles is shown in Figure 2B. At 14 days of age, TUNEL(+) cells were more abundant in both transgenic and non-transgenic *Lama2(−)* muscles, and there was no difference in the total number due to the MyoD-hBcl-2 transgene. At 14 days, all of the *Lama2(−)* muscles examined contained some TUNEL(+) myofibers, however, there was a higher proportion of TUNEL(+) myofibers in MyoD-hBcl-2 transgenic than non-transgenic *Lama2(−)* cohorts (Figures 2A, B). Together, these data are consistent with a delay in initial myofiber apoptosis due to Bcl-2 transgene expression. Here, delayed apoptosis would lead to a lower proportion of TUNEL(+) myofibers in transgenic muscle at 7 days compared with non-transgenic muscle. At 14 days, a higher proportion of TUNEL(+) fibers would be expected in the transgenic tissue as apoptosis progressed, while non-transgenic muscles at 14 days would have proceeded to later events of degeneration and regeneration. Since the Bcl-2 expression was restricted to muscle, no direct effect of the transgene on non-muscle cell apoptosis progression would be expected.

Consistent with prior observations of inflammation in early *Lama2*-deficient muscle pathology [7], we found significant infiltration of CD11b(+) inflammatory cells (macrophages, neutrophils, monocytes) in *Lama2(−)* muscles at both 7 and 14 days. The MyoD-hBcl-2 transgene did not significantly affect the prevalence of CD11b(+) cells at either age (Figure 2C), indicating that inflammatory signals that promote the chemotactic migration of this group of cells were active and not affected by Bcl-2 expression in the muscle cells.

**Figure 3:**
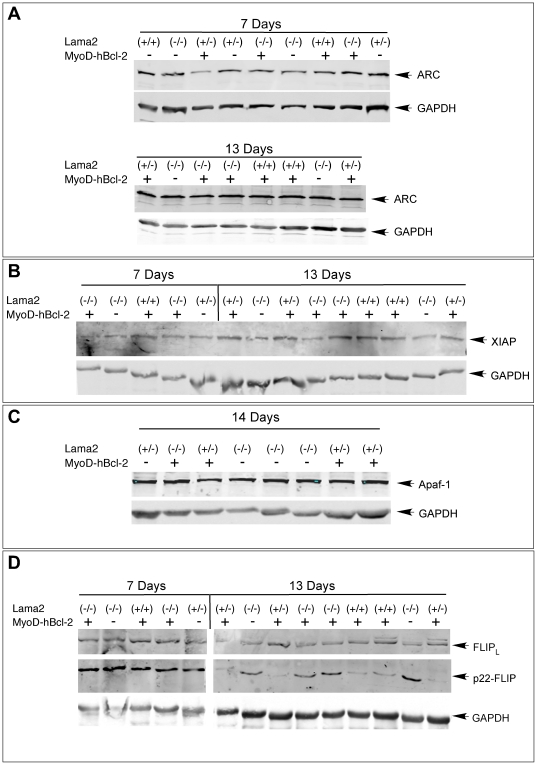
Expression of apoptosis regulators in *Lama2*-deficient muscle. Upper hindlimb muscle proteins from mice of the indicated genotypes and ages were analyzed on western blots probed with antibodies specific to (**A**) ARC, (**B**) XIAP, (**C**) Apaf-1, or (**D**) FLIP. Each blot was also probed with antibodies to GAPDH to normalize for protein loading. ARC, XIAP and Apaf-1, and the 58 kDa FLIP isoform (FLIP_L_, a predominant anti-apoptotic isoform) were present in all muscle samples with no differences associated with *Lama2*-deficiency or the MyoD-hBcl-2 transgene at these ages. The isoform p22-FLIP was expressed in all 7 day-old muscles, but was reduced in *Lama2(+)* muscles at 13 days. *Lama2(−)* muscles maintained a higher level of p22-FLIP at 13 days, and this was not affected by MyoD-hBcl-2 transgene expression.

We examined the expression of several other regulators of apoptosis in these muscles by western blot analysis. The anti-apoptotic proteins ARC, XIAP, or FLIP_L_, and the pro-apoptotic factor Apaf-1 were each present in *Lama2(+)* and *Lama2(−)* muscle and could contribute to muscle survival, but there were no differences in their expression among any of the muscles examined (Figure 3). One exception was the 22 kDa form of FLIP, which was expressed at similar levels in all 7 day-old tissues, then was down regulated in 13 day-old *Lama2(+)* muscle while remaining at higher levels in *Lama2(−)* muscles (Figure 3D). p22-FLIP is a procaspase-8 cleavage product of FLIP that inhibits extrinsic CD95 and TRAIL-induced cell death in immune system cells, but also promotes NF-κβ activation and T cell proliferation [21]. High p22-FLIP levels in day 13 *Lama2^−/−^* muscles might reflect either the presence of proliferating myoblasts participating in muscle regeneration at this age or a role in inhibiting pathology via CD95/TRAIL mechanisms. The Bcl-2 transgene had no effect on its expression.

### Several TLRs are induced early in *Lama2*-deficient muscle pathogenesis

**Figure 4:**
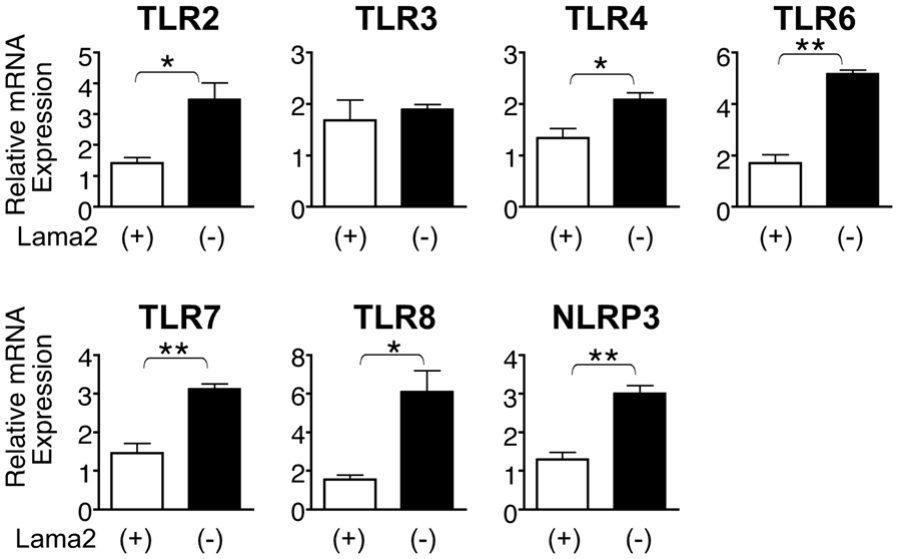
Expression of TLRs and NLRP3 mRNA in 7 day-old *Lama2*-deficient muscles. RNA from upper hindlimb muscles of *Lama2^dy-W^* mice was analyzed by quantitative RT-PCR. *Lama2(−)* muscles expressed significantly higher levels of TLR2, TLR4, TLR6, TLR7, TLR8 and NRLP3 than *Lama2(+)* muscles, while TLR3 was not affected by the absence of Lama2. (n = 4, *p<0.016, **p<0.001).

Our observations that markers of inflammation were elevated within a week after birth suggested that innate immunity mechanisms were activated very early in *Lama2*-deficient muscle disease progression. TLRs are known to function in innate immunity pathways, responding to endogenous pathology-associated ligands as well as exogenous pathogens. We first examined the expression of several TLRs in the *Lama2^dy-W^* parental line at 7 days of age and found that mRNA levels for TLR2, TLR4, TLR6, TLR7 and TLR8, and the inflammasome protein NLRP3 were all significantly higher in upper hindlimb muscles of *Lama2*-deficient mice compared with *Lama2(+)* cohorts. TLR3 expression, however, was unaffected (Figure 4).

### Muscle-specific Bcl-2 inhibits the induction of TLR expression in early *Lama2*-deficient muscle pathogenesis

**Figure 5:**
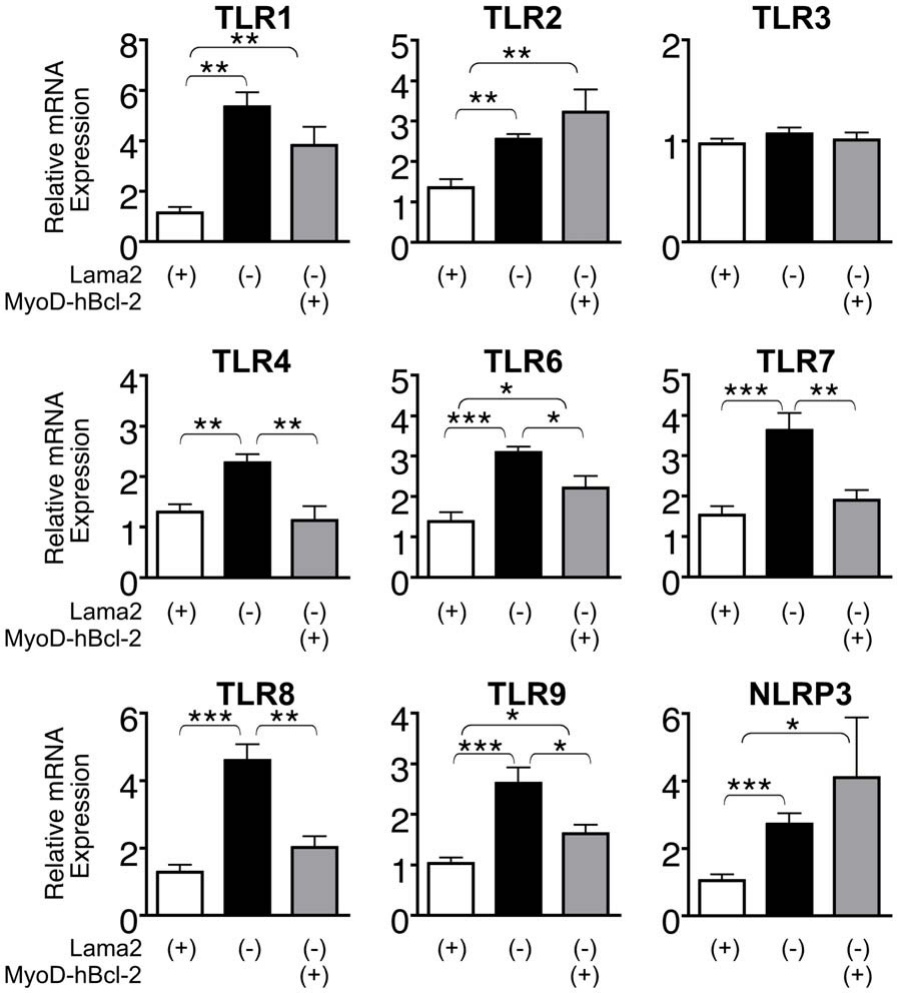
Effects of MyoD-hBcl-2 on expression of TLRs and NLRP3 mRNA in 7 day-old *Lama2*-deficient muscles. RNA from pooled gastrocnemius/soleus muscles was analyzed by quantitative RT-PCR. All TLRs except TLR3, along with NLRP3 were induced in *Lama2(−)* muscles relative to levels in *Lama2(+)* muscles. MyoD-hBcl-2 transgene expression inhibited the induction of TLR4, TLR6, TLR7, TLR8 and TLR9. (n = 5–8, *p<0.05, **p<0.008, ***p<0.0005).

We compared TLR expression in 7 day-old gastrocnemius/soleus muscles from cohorts of MyoD-hBcl-2 transgenic and non-transgenic *Lama2(−)* mice and accompanying *Lama2(+)* controls, all derived by breeding *Lama2^dy-W^*with MyoD-hBcl-2 transgenic mice. As observed in *Lama2^dy-W^* upper hindlimb muscles, mRNAs for NLRP3 and all TLRs (additionally including TLR1 and TLR9) except TLR3 were elevated in *Lama2(−)* gastrocnemius/soleus muscles relative to *Lama2(+)* muscles. These differences in mRNA expression were therefore consistently observed in two different groups of leg muscles and in both the parental mutant *Lama2^dy-W^* mice and mice derived by interbreeding them with the MyoD-hBcl-2 transgenic line. Interestingly, MyoD-hBcl-2 transgene expression in *Lama2(−)* mice resulted in significantly lower levels of TLR4, TLR6, TLR7, TLR8 and TLR9 mRNA (Figure 5). TLR1 was also reduced but not quite to a significant degree and there were no significant transgene-related differences in TLR2, TLR3 or NLRP3. The effect of muscle-specific Bcl-2 expression was greatest on TLR4, TLR7 and TLR8, as their mRNA levels in MyoD-hBcl-2 transgenic *Lama2(−)* muscles remained comparable to those in *Lama2(+)* muscles.

### Muscle-specific Bcl-2 reduces eosinophil infiltration in *Lama2*-deficient muscles

In order to further address the impact of MyoD-hBcl-2 transgene expression on the innate immune response, we examined the levels of eosinophil infiltration in muscles from these 7 day-old mice. Eosinophils are key death-effector cells of innate immune pathways, responding particularly to TLR7-induced proinflammatory signals and drawn to damaged tissues by chemoattractants such as eotaxin-1 (CCL11), a primary chemokine for this cell type. Using wild-type primary myogenic cell cultures, we found that eotaxin-1 mRNA is induced more than 12-fold in differentiated myotubes (but not proliferating myoblasts) treated with the proinflammatory cytokine IFN-γ, and to a smaller degree by IL-4 and IL-6 (Supporting Figure S1, Methods S1). Therefore, differentiated muscle cells can respond to inflammatory cues (e.g. IFN-γ) by synthesizing eotaxin-1, which *in vivo* could promote eosinophil chemotaxis.

**Figure 6:**
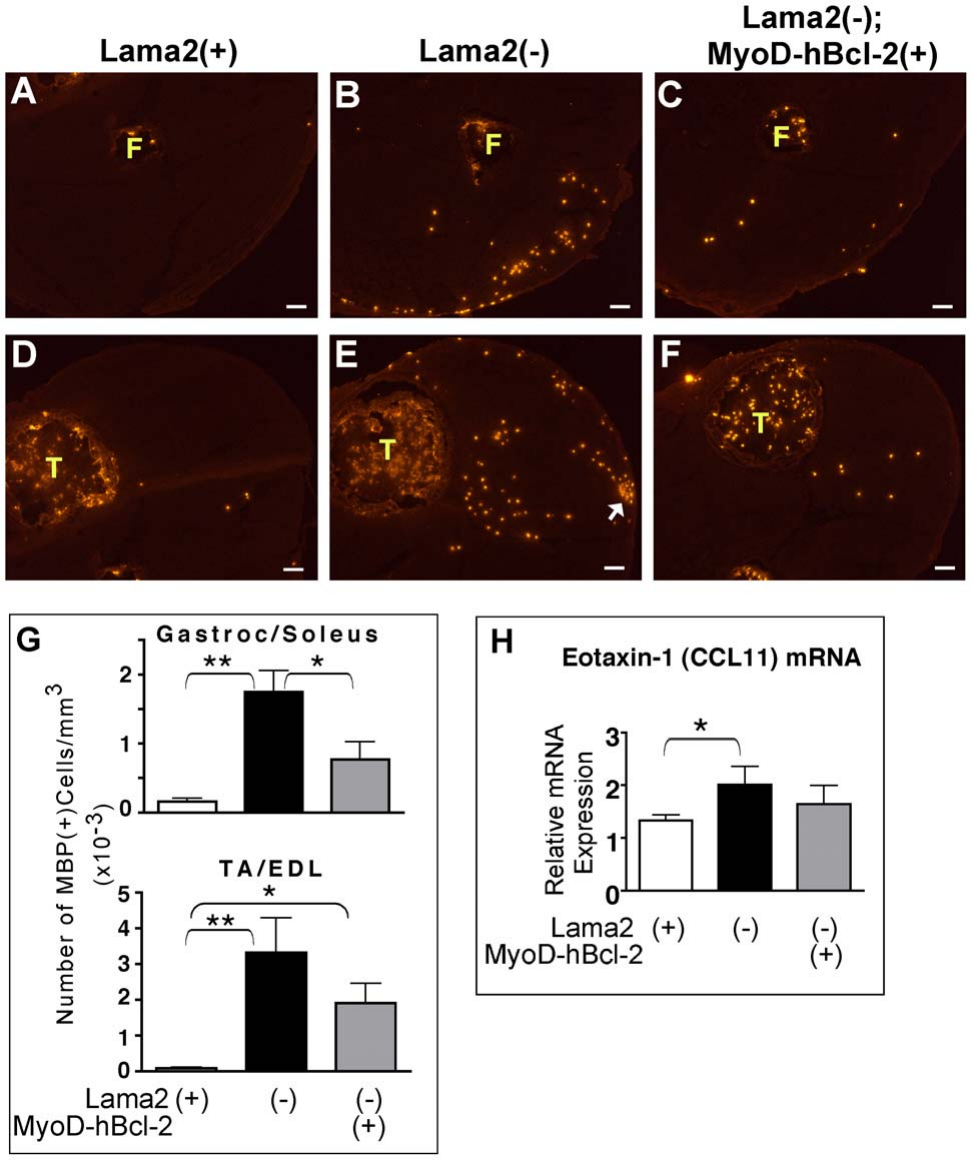
Effects of MyoD-hBcl-2 on eosinophil infiltration and eotaxin-1 expression in 7 day-old *Lama2*-deficient muscles. (**A–F**) Lower hindlimb muscles were immunostained for MBP-1 expression, a marker for eosinophils. Shown are the gastrocnemius/soleus (**A–C**) and TA/EDL (**D–F**) regions from mice of the genotypes indicated. Eosinophil infiltration was evident in *Lama2(−)* muscles, and this was reduced in *Lama2(−);MyoD-hBcl-2(+)* muscles. Arrow indicates a focal cluster of eosinophils often observed in this region of the *Lama2(−)* muscles. (F: fibula and T: tibia, showing eosinophils normally present in bone marrow; scale bars: 100 µM). (**G**) Quantitation of eosinophils in muscle tissues as shown in A–F. (n = 7–13, *p<0.04, **p<0.004). (**H**) Eotaxin-1 mRNA expression in gastrocnemius/soleus muscles was evaluated by quantitative RT-PCR. Eotaxin-1 was induced in *Lama2(−)* muscles compared with *Lama2(+)* controls, but in *Lama2(+);MyoD-hBcl-2* muscles, levels were not significantly higher than *Lama2(+)* controls. (n = 5–8, *p<0.05).

At 7 days of age, significant numbers of eosinophils were present in *Lama2(−)* muscles, with few, if any, in *Lama2(+)* muscles (Figures 6A, B, D, E, G). MyoD-hBcl-2 transgene expression significantly reduced eosinophil numbers in *Lama2(−)* gastrocnemius/soleus muscles (Figures 6B, C, G). A similar trend was observed in tibialis anterior/extensor digitorum longus (TA/EDL) muscles (Figures 6E, F, G) but did not reach statistical significance. This was likely due to higher variability in this region associated with small foci with a high eosinophil density observed in several samples, both with and without the transgene (Figure 6E, arrow). Eotaxin-1 mRNA levels were also elevated in *Lama2(−)* muscles relative to *Lama2(+)* muscles (Figure 6H). Less induction of eotaxin-1 occurred in MyoD-hBcl-2 transgenic *Lama2(−)* muscles, and differences in expression between these and *Lama2(+)* muscles were not significant. Together, these data indicated that muscle-specific expression of Bcl-2 could inhibit synthesis of specific TLRs, chemokine expression (eotaxin-1), and functionally inhibit infiltration of the innate death effector cells (eosinophils) in these diseased neonatal muscles.

## Discussion

Inflammation is an early feature of *Lama2*-deficient muscle, evident by induction of TNFα and IL-1β, elevated TN-C around muscle fibers, inflammatory cell (e.g. macrophage) infiltration and loss of LYVE-1 [7]. Death mechanisms related to the proinflammatory environment present within a week after birth could play a prominent role in the initial muscle loss in this disease. It is therefore important to understand the mechanisms that initiate inflammation in this neonatal period and factors that regulate its progression.

Our data show that some, but not all, of the markers of pathology were reduced in the earliest stages of *Lama2-*deficient disease progression as a result of MyoD-hBcl-2 transgene expression. For example, TN-C was reduced in transgenic *Lama2-*deficient mice at 7 days of age relative to non-transgenic cohorts, but TN-C expression was only delayed, and by 2 weeks no Bcl-2 effects were evident. This muscle-specific Bcl-2 transgene had no effect on the loss of LYVE-1 in vessels within 7 day-old muscle (which likely reflects the presence of a proinflammatory environment based on the sensitivity of this protein to TNFα levels) or the prevalence of CD11b(+) cells (macrophages, neutrophils, monocytes) at either 7 or 14 days. The overall prevalence of apoptotic (TUNEL(+)) cells was also unaffected by the Bcl-2 transgene, although most of these were not muscle cells, and data are consistent with a delay in muscle cell apoptosis in the MyoD-hBcl-2 transgenic compared with non-transgenic *Lama2*-deficient mice.

We show that regulators of innate immunity are induced in these diseased muscles within 7 days after birth, including TLR1, TLR2, TLR4, TLR6, TLR7, TLR8, TLR9, and NLRP3 but not TLR3. Muscle-specific expression of Bcl-2, conferred by a MyoD-hBcl-2 transgene that enhances *Lama2-*deficient mouse long-term survival [19], [20], also affects some of the earliest aspects of disease progression. This includes the novel observation that Bcl-2 expression in *Lama2-*deficient muscle cells inhibits the induction of TLR4, TLR6, TLR7, TLR8 and TLR9, and also reduces the infiltration of eosinophils, which are key death effector cells. Therefore, one or more of the molecular functions of Bcl-2 interact with mechanisms that promote the innate immune response to tissue damage. This early-onset congenital disease model serves as a useful system in which to define these interactions.

In addition to macrophages and other immune system cells where they are best studied, TLRs are also expressed in other cells types. For example, TLR2, TLR4, TLR5 and TLR9 mRNAs are expressed in normal muscle tissue [22]. TLR3 and TLR7 are elevated in inflammatory myopathies [23], [24] as is TLR7 in muscles of Duchenne muscular dystrophy patients [25]. Cultured muscle cells also express mRNAs for TLRs 1–7 and TLR9, and respond to TLR ligands to activate NF-κβ and produce chemokines [22], [23], [24], [26]. Therefore, the elevated levels of TLR and NLRP3 mRNA we observed in *Lama2*-deficient muscles could reflect gene induction either in inflammatory cells (e.g. macrophages) within the tissue or in the muscle cells themselves. Since muscle-specific expression of Bcl-2 resulting from the MyoD-hBcl-2 transgene can prevent the induction of TLR4, TLR6, TLR7, TLR8 and TLR9 in *Lama2*-defient muscle, at least some of these gene expression changes likely occur within the diseased muscle cells themselves.

Of the eight TLRs we examined, only TLR3 was not significantly induced in *Lama2*-deficient muscle. TLR3 signaling is distinct from the other TLRs in that it interacts with the adaptor protein TRIF rather than the adaptor MyD88 (as do all the other TLRs) to activate NF-κβ pathways and induce cytokine production [14], [27]. TLR4 can also interact with TRIF. It will be important to determine whether elements of the MyD88-dependent signaling pathways are involved in the induction of TLRs observed here. The roles of MyD88, TRIF and other TLR adaptors in muscle pathogenesis are currently unknown.

The mechanism by which Bcl-2 prevents the induction of TLR4, TLR6, TLR7, TLR8 and TLR9 is not clear. TLR7, TLR8 and TLR9 are intracellular receptors present on ER membranes in resting cells and are transported to endosomal membranes when activated by nucleic acid ligands [28]. TLR4 on the plasma membrane can also localize to endosomes following ligand stimulation. Bcl-2 is localized at ER membranes where it reduces calcium release from the ER under stress conditions [29], [30]. Bcl-2 could interact with these intracellular TLR receptors at the ER membranes, and somehow modulate their synthesis following activation. TLR4, along with TLR6, is also functional at the plasma membrane (via endosome-independent pathways), as is TLR2, whose expression is not significantly affected by Bcl-2, thus TLR localization does not consistently correlate with Bcl-2 effects on their mRNA levels. In macrophages and other myeloid lineages, TLR transcription is regulated by NF-κβ [31], IFN-β [32] or IFN-γ [33]. Further studies are required to determine whether these factors are involved in regulation of TLR mRNAs in *Lama2*-deficient muscles, and if their levels or activity are modulated directly (or indirectly) by Bcl-2.

Multiple functions of Bcl-2 can inhibit cell death mechanisms [29], [30]. A principal Bcl-2 function is to inhibit intrinsic apoptosis pathways by interacting with pro-apoptotic family members (e.g. Bax/Bak) at mitochondrial membranes, thereby preventing mitochondrial outer membrane permeabilization, release of cytochrome C into the cytoplasm, caspase activation and cell death. As mentioned, Bcl-2 also localizes to ER membranes where it can inhibit cellular death mechanisms involving ER stress and cytotoxic calcium release from the ER. Autophagy can also be inhibited by Bcl-2 via its interactions with Beclin-1, a Bcl-2 family member critical for autophagosome formation. Apoptosis, ER stress and autophagy can each contribute to muscle pathogenesis in muscle diseases, including *Lama2*-deficient and other congenital muscular dystrophies [34], [35], [36], [37]. Further studies are thus needed to determine whether one or more of these mechanisms, or alternatively a novel mechanism, contribute to induction of TLRs in *Lama2*-deficient muscle, and to determine how Bcl-2 expression modulates expression of some but not all TLRs.

Eosinophils, granulocyte effector cells of innate immunity that kill damaged cells or foreign pathogens, were also reduced in Bcl-2 transgenic *Lama2*-deficient muscles compared with non-transgenic cohorts. TLR2 and TLR7 are mediators of eosinophil activation and migration [38]. Our observations support the idea that one or more of these receptors are activated on eosinophils during early phase of muscle pathology and their chemotaxis can be modulated as a downstream consequence of muscle-specific Bcl-2 expression, perhaps via inhibition of eotaxin-1 synthesis. CD11b(+) cell infiltration was not affected by Bcl-2 in transgenic *Lama2*-deficient muscles, therefore only subsets of chemotactic cues might be modulated by its expression.

A key question yet to be answered is: How is the innate immune response initiated by the absence of *Lama2* in diseased muscles? This could involve the presence of a variety of DAMP ligands that activate TLRs leading to the release of IL-1β, TNFα, interferons and other proinflammatory cytokines. For example, the *Lama2*-deficient basal lamina could alter expression of extracellular matrix ligands for TLR2 and TLR4 such as hyaluronan or fibronectin. The ligands HMGB1 or HSPs could be leaked through compromised muscle membranes, as speculated for the innate response observed in dysferlin-deficient muscle [18], [39]. Additionally, intracellular ligands for TLR3, TLR7, TLR8 and TLR9 such as RNA or DNA fragments, possibly derived from apoptotic progression, could exist in *Lama2*-deficient muscle and promote the initial inflammation.

It will be important to determine the mechanisms responsible for TLR and NLRP3 gene activation and the nature of TLR ligands expressed in *Lama2*-deficient muscle that activate pathways of innate immunity. Such information could identify novel points for therapeutic intervention very early in disease progression, which would be of considerable benefit to patients with this severe congenital disease.

## Supporting Information

Figure S1 Expression of eotaxin-1 in muscle cell cultures. Proliferating myoblasts or differentiated myotubes were exposed to the indicated cytokines for 2 or 4 hours then the level of eotaxin-1 mRNA was determined by quantitative RT-PCR. Data is presented as eotaxin-1 mRNA expression relative to the levels in a tumorous spleen (T. Spleen) sample, which served as a strong positive control for this mRNA. Eotaxin-1 was induced in differentiated myotubes exposed to several cytokines, with IFN-γ (IFN) promoting the strongest response.
(TIF)

Methods S1 Cell Culture and Cytokine Treatments.
(DOC)
