# Supplementary figures and images for: Bcl-2 Inhibits the Innate Immune Response during Early Pathogenesis of Murine Congenital Muscular Dystrophy
Source: PLoS One. 2011 Aug 5;6(8):e22369. doi: 10.1371/journal.pone.0022369 (PMC3151242; doi:10.1371/journal.pone.0022369)

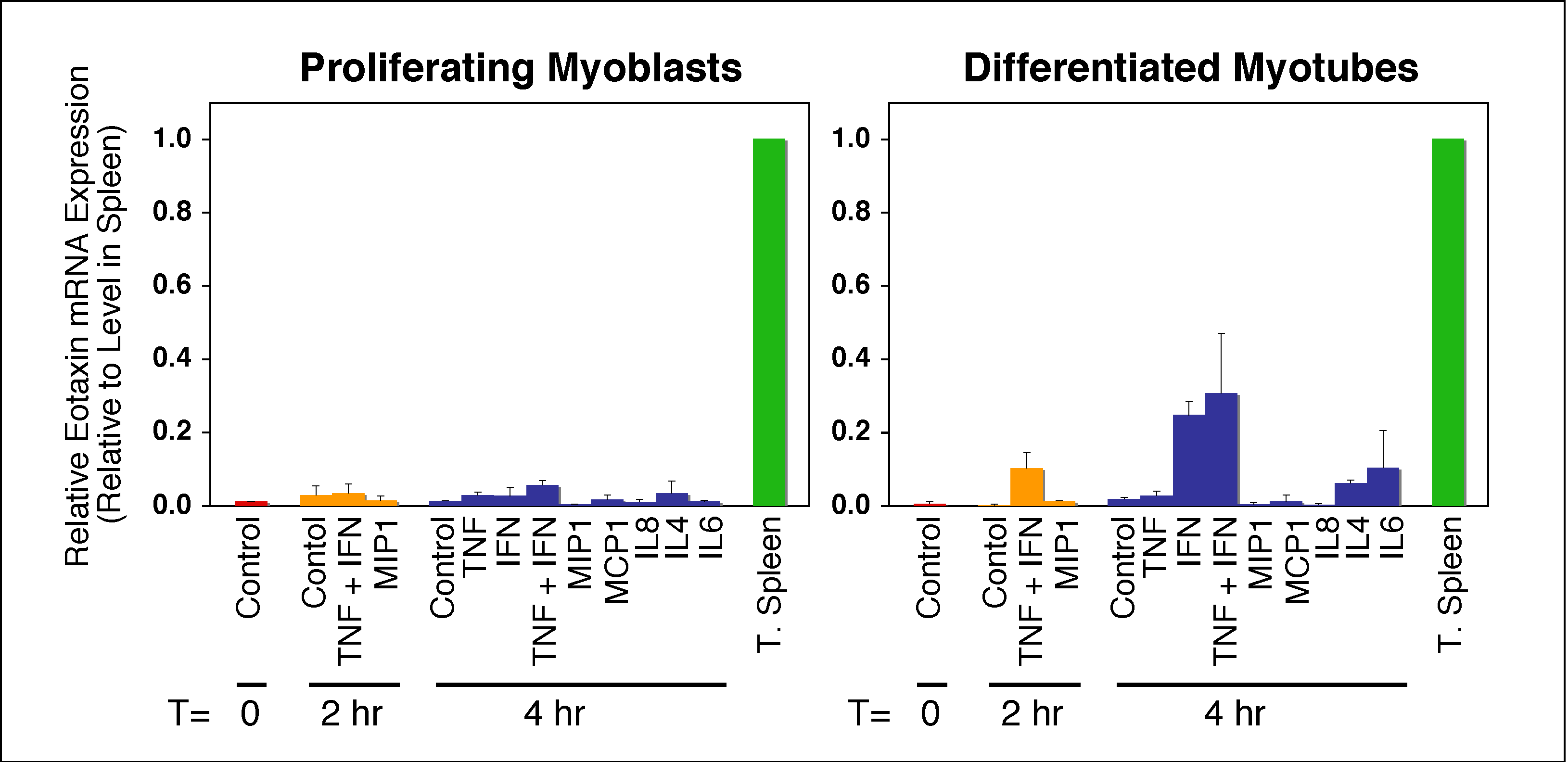

Supplement: Figure S1 — Expression of eotaxin-1 in muscle cell cultures. Proliferating myoblasts or differentiated myotubes were exposed to the indicated cytokines for 2 or 4 hours then the level of eotaxin-1 mRNA was determined by quantitative RT-PCR. Data is presented as eotaxin-1 mRNA expression relative to the levels in a tumorous spleen (T. Spleen) sample, which served as a strong positive control for this mRNA. Eotaxin-1 was induced in differentiated myotubes exposed to several cytokines, with IFN-γ (IFN) promoting the strongest response. (TIF) [file pone.0022369.s001.tif]
