# Supplementary material for: Bcl-2 Inhibits the Innate Immune Response during Early Pathogenesis of Murine Congenital Muscular Dystrophy
Source: PLoS One. 2011 Aug 5;6(8):e22369. doi: 10.1371/journal.pone.0022369 (PMC3151242; doi:10.1371/journal.pone.0022369)
Supplement: Methods S1 — Cell Culture and Cytokine Treatments. (DOC) [file pone.0022369.s002.doc]

**Methods S1. (Supporting Information**)

**Cell Culture and Cytokine Treatments**

Primary myoblasts were isolated from 2 month-old C57BL/6 limb muscles using Percoll gradients to enrich for myogenic cells (typically >95% myogenic cells), and cultured in primary culture medium on E-C-L–coated dishes as previously described [20]. Cells were expanded by passaging twice to generate multiple 35 mm dishes (seeded at 8 x 10^4^ cells/dish). For proliferating myoblast cultures, some of these cells were treated the next day by replacing the culture medium with fresh medium containing various cytokines as listed below. For differentiated myotube cultures, the remaining dishes were fed primary culture medium for an additional 2 days, then medium was replaced with differentiation medium containing 2% horse serum [20] for another 2 days to allow the formation of differentiated myotubes. These differentiated cells then were treated with fresh differentiation medium containing the various cytokines below. For both sets of cells there were duplicate plates for each treatment. Cells were collected just prior to treatment (T=0) and after 2 or 4 hours of treatment, then RNA was analyzed for eotaxin-1 and GAPDH expression by RT-QPCR. RNA from a tumorous spleen that arose spontaneously in a 19 month-old wild-type mouse served as a strong (+) control for eotaxin-1 expression. Cytokines added were: TNFα (200 U/ml), IFN-γ (100 U/ml), combined TNFα (200 U/ml) + IFN-γ (100 U/ml), MIP1α (10 ng/ml), MCP-1 (100 ng/ml), IL-4 (5 ng/ml), IL-6 (5 ng/ml), IL-8 (200 ng/ml), or an equivalent volume of PBS, 0.1% BSA (diluent) as non-treated controls.
